# Poly[bis­(μ_2_-5-{4-[(1*H*-imidazol-1-yl)meth­yl]phen­yl}tetra­zolato)zinc]

**DOI:** 10.1107/S1600536812050672

**Published:** 2013-01-09

**Authors:** Zhe Song

**Affiliations:** a Department of Chemistry, Changchun Normal University, Changchun 130032, People’s Republic of China

## Abstract

In the title compound, [Zn(C_11_H_9_N_6_)_2_]_*n*_, the Zn^II^ atom lies on an inversion center and is coordinated by four N atoms from four 5-[4-(1*H*-imidazol-1-ylmeth­yl)phen­yl]tetra­zolate ligands in a distorted tetra­hedral geometry. The ligands bridge the Zn^II^ atoms, leading to the formation of a two-dimensional network parallel to (010). The structure is further stabilized by C—H⋯N, C—H⋯π and π–π [centroid–centroid distance = 3.7523 (11) Å] inter­actions within the network.

## Related literature

For background to metal-organic architectures, see: Awaleh *et al.* (2005 ▶); Mooibroek & Gamez (2007 ▶); Su *et al.* (2009 ▶). For background to metal–azolate frameworks, see: Darling *et al.* (2012 ▶). For related structures, see: Huang *et al.* (2009 ▶); Su *et al.* (2009 ▶).
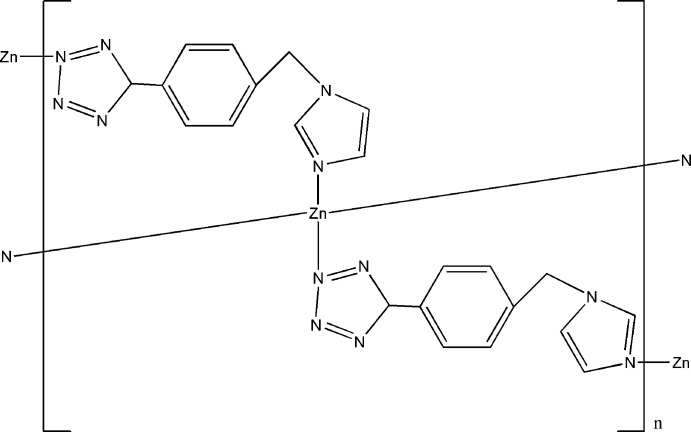


## Experimental

### 

#### Crystal data

[Zn(C_11_H_9_N_6_)_2_]
*M* *_r_* = 515.85
Orthorhombic, 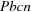

*a* = 16.1206 (12) Å
*b* = 9.3720 (7) Å
*c* = 14.6367 (11) Å
*V* = 2211.3 (3) Å^3^
*Z* = 4
Mo *K*α radiation
μ = 1.15 mm^−1^
*T* = 273 K
0.28 × 0.26 × 0.24 mm

#### Data collection

Bruker APEXII CCD area-detector diffractometer
Absorption correction: multi-scan (*SADABS*; Bruker, 2001 ▶) *T*_min_ = 0.736, *T*_max_ = 0.752
11210 measured reflections
2105 independent reflections
1874 reflections with *I* > 2σ(*I*)
*R*_int_ = 0.020

#### Refinement

*R*[*F* ^2^ > 2σ(*F* ^2^)] = 0.029
*wR*(*F* ^2^) = 0.081
*S* = 1.07
2105 reflections
159 parameters
H-atom parameters constrained
Δρ_max_ = 0.29 e Å^−3^
Δρ_min_ = −0.27 e Å^−3^

### 

Data collection: *APEX2* (Bruker, 2007 ▶); cell refinement: *SAINT* (Bruker, 2007 ▶); data reduction: *SAINT*; program(s) used to solve structure: *SHELXTL* (Sheldrick, 2008 ▶); program(s) used to refine structure: *SHELXTL*; molecular graphics: *XP* in *SHELXTL* and *DIAMOND* (Brandenburg, 1999 ▶); software used to prepare material for publication: *SHELXTL*.

## Supplementary Material

Crystal structure: contains datablock(s) I, global. DOI: 10.1107/S1600536812050672/zq2192sup1.cif

Structure factors: contains datablock(s) I. DOI: 10.1107/S1600536812050672/zq2192Isup2.hkl

Supplementary material file. DOI: 10.1107/S1600536812050672/zq2192Isup3.cdx

Additional supplementary materials:  crystallographic information; 3D view; checkCIF report

## Figures and Tables

**Table 1:** Hydrogen-bond geometry (Å, °) *Cg*2 is the centroid of the C1–C6 ring.

*D*—H⋯*A*	*D*—H	H⋯*A*	*D*⋯*A*	*D*—H⋯*A*
C10—H10⋯N4^i^	0.93	2.45	3.344 (2)	163
C8—H8*A*⋯*Cg*2^ii^	0.97	2.88	3.692 (2)	142

Symmetry codes: (i) 
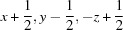
; (ii) 
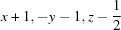
.

**Fig. 1.**
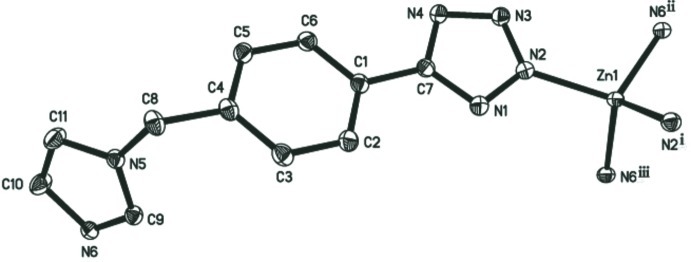
The asymmetric unit of the title compound. Displacement ellipsoids are drawn at the 30% probability level. H atoms have been omitted for clarity. Symmetry codes: (i) -x+1, y, -z+3/2; (ii) -x+3/2, -y-1/2, z+1/2; (iii) x-1/2, -y-1/2, -z+1.

**Fig. 2.**
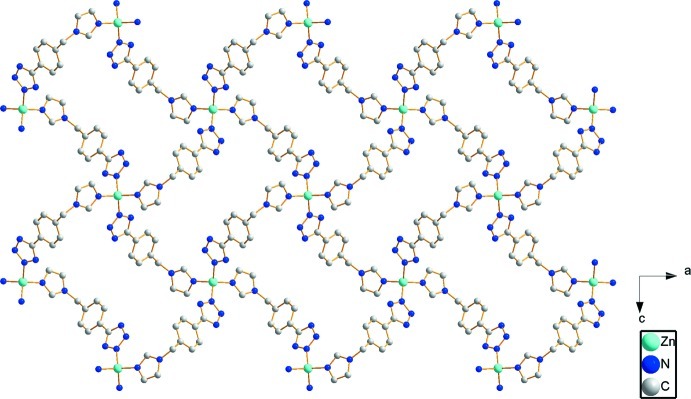
View of the two-dimensional network of the title compound.

## supplementary crystallographic information

### Comment

Metal–Organic Frameworks (MOFs) continue to receive significant contemporary attention, reflecting their applications to fields as diverse as gas storage, separation, and catalysis (Mooibroek *et al.*, 2007; Awaleh *et al.*, 2005). Transition metal complexes using tetrazole derivatives as ligands are of great interest as many compounds based on these ligands have shown intriguing structures with interesting properties (Su *et al.*, 2009; Darling *et al.*, 2012). Recently, we obtained the title complex by the reaction of zincacetate with 5-(4-imidazol-1-yl-benzyl)-2*H*-tetrazole using hydrothermal method and its crystal structure is reported here.

In the title compound, the Zn^II^ atom lies on an inversion center and adopts a distorted tetrahedral coordination geometry, being coordinated by four N atoms from four azolate ligands (Fig. 1). The bridging azolate ligands allow the formation of a two-dimensional network parallel to (010) (Fig. 2), while in a related structure the azolate C_11_H_9_N_6_ ligands form one-dimensional chains with the Zn^II^ atoms (Huang *et al.*, 2009). The crystal structure is further stabilized by C–H···N, C–H···π and and π-π interactions within the network (see Geometric parameters and Table 1: Cg1 and Cg2 corresponding to the centroids of the N1-N2-N3-N4-C7 and C1–C6 rings, respectively).

### Experimental

A mixture of Zn(OAc)_2_.2H_2_O (0.2 mmol, 0.043 g), 5-(4-imidazol-1-yl-benzyl)-2*H*-tetrazole (0.2 mmol, 0.045 g), NH_3_.H_2_O (2 mL), EtOH (5 ml) and water (5 ml) was sealed in a 15 ml Teflon-lined reactor, which was heated at 100°C for 72 h and then gradually cooled to room temperature. Colourless crystals were obtained.

### Refinement

The H atoms were generated geometrically and refined as riding atoms, with C—H = 0.93 (aromatic) or 0.97 (CH_2_) Å and *U*_iso_(H) = 1.2*U*_eq_(C).

### Crystal data

**Table:**

[Zn(C_11_H_9_N_6_)_2_]	*F*(000) = 1056
*M**_r_* = 515.85	*D*_x_ = 1.549 Mg m^−^^3^
Orthorhombic, *P**b**c**n*	Mo *K*α radiation, λ = 0.71073 Å
Hall symbol: -P 2n 2ab	Cell parameters from 2105 reflections
*a* = 16.1206 (12) Å	θ = 2.5–25.7°
*b* = 9.3720 (7) Å	µ = 1.15 mm^−^^1^
*c* = 14.6367 (11) Å	*T* = 273 K
*V* = 2211.3 (3) Å^3^	Block, colourless
*Z* = 4	0.28 × 0.26 × 0.24 mm

### Data collection

**Table:**

Bruker APEXII CCD area-detector diffractometer	2105 independent reflections
Radiation source: fine-focus sealed tube	1874 reflections with *I* > 2σ(*I*)
Graphite monochromator	*R*_int_ = 0.020
phi and ω scans	θ_max_ = 25.7°, θ_min_ = 2.5°
Absorption correction: multi-scan (*SADABS*; Bruker, 2001)	*h* = −13→19
*T*_min_ = 0.736, *T*_max_ = 0.752	*k* = −11→11
11210 measured reflections	*l* = −17→15

### Refinement

**Table:**

Refinement on *F*^2^	Primary atom site location: structure-invariant direct methods
Least-squares matrix: full	Secondary atom site location: difference Fourier map
*R*[*F*^2^ > 2σ(*F*^2^)] = 0.029	Hydrogen site location: inferred from neighbouring sites
*wR*(*F*^2^) = 0.081	H-atom parameters constrained
*S* = 1.07	*w* = 1/[σ^2^(*F*_o_^2^) + (0.045*P*)^2^ + 1.2542*P*] where *P* = (*F*_o_^2^ + 2*F*_c_^2^)/3
2105 reflections	(Δ/σ)_max_ < 0.001
159 parameters	Δρ_max_ = 0.29 e Å^−^^3^
0 restraints	Δρ_min_ = −0.27 e Å^−^^3^

### Special details

**Table:**

Geometry. All e.s.d.'s (except the e.s.d. in the dihedral angle between two l.s. planes) are estimated using the full covariance matrix. The cell e.s.d.'s are taken into account individually in the estimation of e.s.d.'s in distances, angles and torsion angles; correlations between e.s.d.'s in cell parameters are only used when they are defined by crystal symmetry. An approximate (isotropic) treatment of cell e.s.d.'s is used for estimating e.s.d.'s involving l.s. planes.
Refinement. Refinement of *F*^2^ against ALL reflections. The weighted *R*-factor *wR* and goodness of fit *S* are based on *F*^2^, conventional *R*-factors *R* are based on *F*, with *F* set to zero for negative *F*^2^. The threshold expression of *F*^2^ > σ(*F*^2^) is used only for calculating *R*-factors(gt) *etc*. and is not relevant to the choice of reflections for refinement. *R*-factors based on *F*^2^ are statistically about twice as large as those based on *F*, and *R*- factors based on ALL data will be even larger.

### Fractional atomic coordinates and isotropic or equivalent isotropic displacement parameters (Å^2^)

**Table:**

	*x*	*y*	*z*	*U*_iso_*/*U*_eq_	
Zn1	0.5000	−0.00874 (3)	0.7500	0.02093 (12)	
N1	0.56706 (10)	−0.22486 (17)	0.62224 (10)	0.0287 (4)	
N2	0.50779 (9)	−0.12643 (17)	0.63732 (11)	0.0250 (3)	
N3	0.45760 (9)	−0.11431 (16)	0.56620 (10)	0.0258 (3)	
N4	0.48338 (10)	−0.20502 (19)	0.50222 (9)	0.0251 (4)	
N5	0.78108 (9)	−0.70455 (15)	0.30116 (10)	0.0227 (3)	
N6	0.89739 (10)	−0.60890 (17)	0.25437 (9)	0.0236 (3)	
C1	0.59424 (12)	−0.3877 (2)	0.49151 (11)	0.0243 (4)	
C2	0.65678 (12)	−0.4625 (2)	0.53566 (13)	0.0294 (4)	
H2	0.6729	−0.4359	0.5942	0.080*	
C3	0.69536 (13)	−0.5767 (2)	0.49300 (12)	0.0300 (4)	
H3	0.7372	−0.6260	0.5232	0.080*	
C4	0.67212 (11)	−0.61812 (19)	0.40553 (12)	0.0258 (4)	
C5	0.61043 (12)	−0.5417 (2)	0.36076 (13)	0.0277 (4)	
H5	0.5949	−0.5677	0.3019	0.080*	
C6	0.57190 (11)	−0.4272 (2)	0.40282 (12)	0.0271 (4)	
H6	0.5310	−0.3764	0.3719	0.080*	
C7	0.54942 (11)	−0.27135 (19)	0.53796 (12)	0.0234 (4)	
C8	0.71159 (12)	−0.74616 (19)	0.36103 (13)	0.0299 (4)	
H8A	0.7317	−0.8105	0.4079	0.080*	
H8B	0.6702	−0.7966	0.3253	0.080*	
C9	0.84655 (11)	−0.62443 (18)	0.32411 (12)	0.0233 (4)	
H9	0.8550	−0.5850	0.3817	0.080*	
C10	0.86280 (13)	−0.6845 (2)	0.18321 (13)	0.0370 (5)	
H10	0.8853	−0.6934	0.1250	0.080*	
C11	0.79073 (13)	−0.7436 (2)	0.21178 (14)	0.0360 (5)	
H11	0.7548	−0.7997	0.1774	0.080*	

### Atomic displacement parameters (Å^2^)

**Table:**

	*U*^11^	*U*^22^	*U*^33^	*U*^12^	*U*^13^	*U*^23^
Zn1	0.01744 (19)	0.02404 (18)	0.02131 (19)	0.000	−0.00210 (10)	0.000
N1	0.0271 (8)	0.0325 (8)	0.0265 (8)	0.0039 (7)	−0.0012 (6)	−0.0046 (7)
N2	0.0234 (8)	0.0271 (8)	0.0245 (8)	0.0004 (6)	−0.0012 (6)	−0.0019 (7)
N3	0.0253 (8)	0.0272 (8)	0.0249 (8)	−0.0019 (6)	0.0007 (6)	−0.0013 (6)
N4	0.0259 (8)	0.0277 (9)	0.0218 (8)	−0.0003 (7)	0.0010 (6)	−0.0013 (6)
N5	0.0204 (7)	0.0243 (7)	0.0234 (8)	−0.0017 (6)	0.0044 (6)	−0.0008 (6)
N6	0.0203 (8)	0.0265 (8)	0.0239 (8)	−0.0002 (6)	0.0025 (6)	−0.0012 (6)
C1	0.0226 (9)	0.0261 (9)	0.0241 (9)	−0.0036 (7)	0.0054 (7)	0.0004 (7)
C2	0.0286 (10)	0.0357 (10)	0.0241 (10)	0.0017 (8)	0.0021 (8)	−0.0008 (8)
C3	0.0280 (10)	0.0334 (11)	0.0286 (10)	0.0037 (8)	0.0039 (8)	0.0040 (8)
C4	0.0237 (9)	0.0246 (9)	0.0290 (9)	−0.0034 (7)	0.0096 (7)	0.0022 (7)
C5	0.0269 (10)	0.0309 (9)	0.0254 (9)	−0.0039 (8)	0.0037 (8)	−0.0030 (8)
C6	0.0250 (9)	0.0299 (10)	0.0264 (9)	−0.0009 (8)	0.0013 (8)	0.0006 (8)
C7	0.0220 (9)	0.0250 (9)	0.0231 (9)	−0.0031 (7)	0.0026 (7)	0.0008 (7)
C8	0.0276 (10)	0.0259 (9)	0.0361 (10)	−0.0030 (7)	0.0135 (8)	0.0007 (8)
C9	0.0236 (9)	0.0237 (9)	0.0228 (9)	−0.0015 (7)	0.0025 (7)	−0.0009 (7)
C10	0.0322 (11)	0.0568 (13)	0.0221 (9)	−0.0122 (10)	0.0048 (8)	−0.0085 (9)
C11	0.0318 (11)	0.0515 (13)	0.0246 (10)	−0.0116 (9)	0.0022 (8)	−0.0101 (9)

### Geometric parameters (Å, º)

**Table:**

Zn1—N2	1.9880 (16)	C1—C7	1.474 (3)
Zn1—N2^i^	1.9880 (16)	C2—C3	1.386 (3)
Zn1—N6^ii^	1.9889 (16)	C2—H2	0.9300
Zn1—N6^iii^	1.9889 (16)	C3—C4	1.390 (3)
N1—C7	1.339 (2)	C3—H3	0.9300
N1—N2	1.346 (2)	C4—C5	1.390 (3)
N2—N3	1.323 (2)	C4—C8	1.506 (2)
N3—N4	1.331 (2)	C5—C6	1.384 (3)
N4—C7	1.339 (2)	C5—H5	0.9300
N5—C9	1.338 (2)	C6—H6	0.9300
N5—C11	1.367 (2)	C8—H8A	0.9700
N5—C8	1.475 (2)	C8—H8B	0.9700
N6—C9	1.317 (2)	C9—H9	0.9300
N6—C10	1.377 (2)	C10—C11	1.353 (3)
N6—Zn1^iv^	1.9889 (16)	C10—H10	0.9300
C1—C2	1.388 (3)	C11—H11	0.9300
C1—C6	1.397 (2)	Cg1—Cg2^v^	3.7523 (11)
			
N2—Zn1—N2^i^	112.61 (9)	C3—C4—C5	118.89 (17)
N2—Zn1—N6^ii^	106.36 (6)	C3—C4—C8	120.48 (17)
N2^i^—Zn1—N6^ii^	109.48 (6)	C5—C4—C8	120.61 (17)
N2—Zn1—N6^iii^	109.48 (6)	C6—C5—C4	120.74 (17)
N2^i^—Zn1—N6^iii^	106.36 (6)	C6—C5—H5	119.6
N6^ii^—Zn1—N6^iii^	112.67 (9)	C4—C5—H5	119.6
C7—N1—N2	102.90 (15)	C5—C6—C1	120.20 (18)
N3—N2—N1	111.33 (14)	C5—C6—H6	119.9
N3—N2—Zn1	124.49 (12)	C1—C6—H6	119.9
N1—N2—Zn1	124.13 (12)	N1—C7—N4	112.20 (16)
N2—N3—N4	107.92 (14)	N1—C7—C1	124.16 (17)
N3—N4—C7	105.65 (14)	N4—C7—C1	123.56 (16)
C9—N5—C11	107.49 (14)	N5—C8—C4	111.54 (14)
C9—N5—C8	126.75 (15)	N5—C8—H8A	109.3
C11—N5—C8	125.75 (15)	C4—C8—H8A	109.3
C9—N6—C10	106.10 (15)	N5—C8—H8B	109.3
C9—N6—Zn1^iv^	127.13 (12)	C4—C8—H8B	109.3
C10—N6—Zn1^iv^	126.68 (12)	H8A—C8—H8B	108.0
C2—C1—C6	119.07 (17)	N6—C9—N5	110.99 (15)
C2—C1—C7	121.01 (16)	N6—C9—H9	124.5
C6—C1—C7	119.88 (17)	N5—C9—H9	124.5
C3—C2—C1	120.42 (18)	C11—C10—N6	108.92 (16)
C3—C2—H2	119.8	C11—C10—H10	125.5
C1—C2—H2	119.8	N6—C10—H10	125.5
C2—C3—C4	120.66 (18)	C10—C11—N5	106.49 (16)
C2—C3—H3	119.7	C10—C11—H11	126.8
C4—C3—H3	119.7	N5—C11—H11	126.8

Symmetry codes: (i) −*x*+1, *y*, −*z*+3/2; (ii) −*x*+3/2, −*y*−1/2, *z*+1/2; (iii) *x*−1/2, −*y*−1/2, −*z*+1; (iv) −*x*+3/2, −*y*−1/2, *z*−1/2; (v) −*x*+1, −*y*−1, −*z*+1.

### Hydrogen-bond geometry (Å, º)

Cg2 is the centroid of the C1–C6 ring.

**Table:**

*D*—H···*A*	*D*—H	H···*A*	*D*···*A*	*D*—H···*A*
C10—H10···N4^vi^	0.93	2.45	3.344 (2)	163
C8—H8*A*···*Cg*2^vii^	0.97	2.88	3.692 (2)	142

Symmetry codes: (vi) *x*+1/2, *y*−1/2, −*z*+1/2; (vii) *x*+1, −*y*−1, *z*−1/2.
